# Supplementary material for: Ca2+ signaling driving pacemaker activity in submucosal interstitial cells of Cajal in the murine colon
Source: eLife. 2021 Jan 5;10:e64099. doi: 10.7554/eLife.64099 (PMC7806270; doi:10.7554/eLife.64099)
Supplement: Supplementary file 1. [file elife-64099-supp1.docx]

| **Gene** | **Primer Sequence** | **GenBank Accession Number** |
| --- | --- | --- |
| *Kit* | \| F-CGCCTGCCGAAATGTATGACG \| \| --- \| \| R-GGTTCTCTGGGTTGGGGTTGC \| | NM_021099 |
| *Ano1* | \| F-TAACCCTGCCACCGTCTTCT \| \| --- \| \| R-ATGATCCTTGACAGCTTCCTCC \| | NM_178642 |
| *Myh11* | \| F-CCCAAGCAGCTAAAGGACAA \| \| --- \| \| R-AGGCACTTGCATTGTAGTCC \| \| | NM_013607 |
| *Uchl1* | \| F-CGATGGAGATTAACCCCGAGATG \| \| --- \| \| R-TTTTCATGCTGGGCCGTGAG \| | NM_011670 |
| *Cacna1c* | \| F-GTAAGGATGAGTGAAGAAGCCGAGTAC \| \| --- \| \| R-CAGAGCGAAGGAAACTCCTCTTTGG \| | NM_009781 |
| *Cacna1d* | \| F-ACCAAAGAAACAGAAGGCGG \| \| --- \| \| R-TGTAAACTGGGCACTCCTGA \| \| | NM_028981 |
| *Cacna1g* | \| F-ACAACGGCATGGCCTCCACGT \| \| --- \| \| R-CCGTTTGCCGATTTCCTCTGCCTG \| | NM_021415 |
| *Cacna1h* | \| F-TGGAGACCTACACAGGCCCGGT \| \| --- \| \| R-CAGAGAGCGGGGCGTATCC \| | NM_001044308 |
| *Orai1* | \| F-GTTCACTTCTACCGCTCCCT \| \| --- \| \| R-GTGCCCGGTGTTAGAGAATG \| | NM_175423 |
| *Orai2* | \| F-CACAAGGGCATGGATTACCG \| \| --- \| \| R-CCCTGCTCAGGTAGAGCTTC \| | NM_178751 |
| *Orai3* | \| F-GGCTGAAGTTGTTCTGGTGG \| \| --- \| \| R-TGGAAGGCTGTTGTGATGTG \| | NM_198424 |
|  |  |  |
